
JOURNAL INFORMATION
==============================
NLM Title Abbreviation: Ann R Coll Surg Engl
Journal ID: Ann R Coll Surg Engl
NLM Journal ID: 7506860
Journal ID: ann

Annals of The Royal College of Surgeons of England

ISSN: 0035-8843
EISSN: 1478-7083
Publisher: Royal College of Surgeons of England

ARTICLE INFORMATION
==============================
PMCID: PMC12934898
DOI: 10.1308/rcsann.2026.0029
Article ID: rcsann.2026.0029
Article version: 1
Subjects: ASiT Innovation Summit E-Posters

ASiT Innovation Summit E-Posters

Print publication date: 2026 Feb 25
Volume: 108
Issue: Suppl 1
First page: S18
Last page: S64
Copyright: Copyright © 2026, The Authors
Copyright year: 2026
License: This is an open-access article distributed under the terms of the Creative Commons Attribution 4.0 International License, which permits unrestricted use, distribution, reproduction, and adaptation in any medium, provided the original work is properly attributed.
License URL: https://creativecommons.org/licenses/by/4.0/

==============================

Subjects: Artificial Intelligence in Surgery

6 Enhancing ENT induction with conversational avatars: improving confidence and satisfaction in epistaxis care

Qamar Amna
Maweni Robert
Oxford University Hospitals NHS Trust, Oxford, UK

7 How have generic large language models progressed in their ability to write clinical letters and manage patients in the virtual fracture clinic?

Smith Amy
Brock James
Anss Rana
Kimberley Charles
Joyner Claire
Solari Francesca
Yasin Tariq
Poacher Arwel
University Hospital of Wales, Cardiff, UK

39 The perfect AI research assistant: are ScholarGPT references more reliable than ChatGPT references?

Tulimat Bisher 1
Kiwan Omar 2
Al-Kalbani Mohammed 2
Musa Amr 1
Edlebi Omar 1
Munro Tyler 2
1 Royal College of Surgeons in Ireland, MUB, Busaiteen, Bahrain
2 University of Manchester, Manchester, UK

52 The role of artificial intelligence and machine learning applications in emergency surgery: a systematic review of diagnostic accuracy and clinical outcomes

Baqar Safa 1
Hamed Adel 2
Elsaiegh Mohammed 3
1 Royal Free London NHS Trust, London, UK
2 Aneurin Bevan University Health Board, Newport, UK
3 Royal Cornwall Hospital, Truro, UK

93 AI-assisted cystoscopy for bladder cancer: current evidence and clinical potential (2020–2025)

Alsharief Mazen
Alawadi Abdullah
University Hospitals Birmingham, Birmingham, UK

94 Risk-stratification machine learning (AI) models for microscopic haematuria (2020–2025)

Alsharief Mazen
Alawadi Abdullah
University Hospitals Birmingham, Birmingham, UK

172 Bias in surgical artificial intelligence models: a systematic review of algorithmic fairness and surgical outcome

Tsang Marcus Owen 1
Leung Yiu Ka Nathaniel 2
1 Newcastle University, Newcastle Upon Tyne, UK
2 Queen’s University Belfast, Belfast, UK

199 BASSGPT: a grounded retrieval augmented generation large language model for spine surgery patients

Biswas Sayan 1
Sarkar Ved 2
George K. Joshi 1
1 Manchester Centre for Clinical Neurosciences, Manchester, UK
2 University of California Berkeley, Berkeley, USA

213 Evaluating ChatGPT-4's performance on the FRCS urology part a examination

Kafagi Abdul-Hadi 1
Kafagi Abdul Rhaman 2
1 Manchester NHS Foundation Trust, Manchester, UK
2 University of Manchester, Manchester, UK

462 Intraoperative AI-enhanced optical/spectroscopic imaging in glioma surgery: a pooled analysis

Sunny Jesvin T 1
Manoharasundaram Kriisan 1
Ebrahimian-Roodbari Neima 1
Kalaga Sreevishnu 1
Lam Kalista 2
Tsang Kevin 1
1 Imperial College Healthcare NHS Trust, London, UK
2 Imperial College London, London, UK

Subjects: Clinical Research

3 Trans-nasal oesophagoscopy: does it have a role in diagnosing malignant disease?

Larwood Jessica
East and North Hertfordshire NHS Trust, Stevenage, UK

8 Mesorectal nodal threat to the circumferential resection margin predicts outcomes comparable to tumour-related CRM involvement in rectal cancer

Zin May Thu 1
Coker Oluwafemi 2
Rais Khurram 1
Alam Beenish 1
Saleem Shamsa 1
Ray Christopher 1
1 University Hospital Crosshouse, NHS Ayrshire and Arran, Kilmarnock, UK
2 Queen Elizabeth Hospital, NHS Greater Glasgow and Clyde, Glasgow, UK

22 Long-term angiographic outcomes and clinical durability of endovascular treatment for indirect carotid–cavernous fistulas: a retrospective study

Saghebdoust Sajjad 1 2 3
Kheradmand Daniel 2
Zabihyan Samira 2
Mirbolouk Mohammad Hossein 2
Baharvahdat Humain 4 2 3
1 Department of Neurosurgery, Queen's Hospital, Barking Havering and Redbrige University Hospitals NHS Trust, Romford, UK
2 Neuroendovascular Section, Department of Neurosurgery, Ghaem Hospital, Mashhad University of Medical Sciences, Mashhad, Iran, Islamic Republic of
3 Department of Neurosurgery, Razavi Hospita, Mashhad, Iran, Islamic Republic of
4 Department of Interventional Neuroradiology, Rothschild Foundation Hospital, Parice, France

31 Does the timing of ankle fracture surgery influence post-operative length of stay (LoS) and surgical complications

Raza Farwa
Kong Roderick
Hazarika Shariff
Royal Alexandra Hospital, Glasgow, UK

48 A prospective observational study comparing retrorectus and anterectus mesh placement in incisional hernia repair

Bakka Havil Stephen Alexander 1
Kamepalli Prathibha 2
Varathan Kayaththery 3
Zacken Adele 4
Kirupakaran Tharaga 5
Koshy Daniel I. 6
Bharadwaj Sanjeevi 7
1 Royal Sussex County Hospital, Brighton, UK
2 NRI Medical College & General Hospital, Vijayawada, India
3 Barts Health NHS Trust, London, UK
4 Havering and Redbridge University Hospitals NHS Trust, London, UK
5 Kingston Hospital NHS Foundation Trust, Kingston, UK
6 Royal London Hospital, London, UK
7 National Health Service (NHS), Birmingham, UK

95 Critical review of ICD-9 to ICD-10 system-based complication codes for measuring postoperative surgical complications using large administrative datasets

Kumar Radhe Shantha 1
Uribe-Leitz Tarsicio 2
Molina George 2
Adler Rachel 2
Clark Clancy J. 3
1 University of Manchester, Manchester, UK
2 Center for Surgery and Public Health, Brigham and Women’s Hospital, Boston, USA
3 Department of General Surgery, Virginia Mason Medical Center, Seattle, USA

102 Comparison of short-term clinical outcomes between robotic, minimally invasive and open esophagectomy in esophageal cancer surgery: systematic review

Varathan Kayaththery 1
Zacken Adele 2
Ravichandran Praveen Surya 2
Bakka Havil Stephen Alexander 3
Albayati Mustafa 1
Khan Uzair 4
1 Department of Trauma and Orthopaedics, Barts Health NHS Trust, London, UK
2 Department of General Surgery, Barts Health NHS Trust, London, UK
3 Department of Neurosurgery, University Hospital Sussex NHS Trus, Brighton, UK
4 University of Cambridge, Cambridge, UK

104 Content and face validity of a novel homemade laparoscope and laparoscopic camera navigation model: a pilot study

Varathan Kayaththery 1
Zacken Adele 2
Bakka Havil Stephen Alexander 3
Kirupakaran Tharaga 4
Albayati Mustafa 1
Khan Uzair 5
Bharadwaj Sanjeevi 1
1 Department of Trauma and Orthopaedics, Barts Health NHS Trust, London, UK
2 Department of General Surgery, Barts Health NHS Trust, London, UK
3 Department of Neurosurgery, University Hospital Sussex NHS Trust, Brighton, UK
4 4 Department of Trauma and Orthopaedics, Epsom and St. Helier NHS Trust, Sutton, UK
5 University of Cambridge, Cambridge, UK

134 Evaluation of high ligation operation in varicocele as regard to semen parameters, testicular functions and postoperative complications

Abdelnour David Safwat Thabet 1 2
Mahfouz Mohamed 1
Boshra Wadie 1
Farag Peter 2
1 Ain Shams University, Cairo, Egypt
2 Kettering General hospital “Current”, Kettering Northamptonshire, UK

228 Trends in the microbiological profile of prosthetic joint infections: are pathogens getting more sophisticated?

Oladeji Emmanuel
Okonkwo Patrick
Omerenma Onyeka
Alhashash Mohammad
Obakponovwe Oghofori
St Richards Hospital, Chichester, UK

250 Laparoscopy: a comprehensive approach for diagnosis and treatment of abdominal pain

Maula Kulsum 1
Rahman Md Mustafizur 2
Khan Mohammad Emrul Hasan 3
Hasan Md Nazmul 4
Ahmed Md Nadim 2
1 East Suffolk and North Essex NHS foundation Trust, Colchester, UK
2 Shaheed Suhrawardy Medical College Hospital, Dhaka, Bangladesh
3 Dhaka Medical Collage Hospital, Dhaka, Bangladesh
4 Labaid Specialised Hospital, Dhaka, Bangladesh

266 Prevalence, characterisation, and clinical impact of anaemia in patients with intermittent claudication: a retrospective analysis

Hong Jae Seon 1
Loy Grace 1
Sivaharan Ashwin 1
Sillito Sarah 1
Nandhra Sandip 1 2
El-Sayed Tamer 1
1 The Northern Vascular Centre, Newcastle upon Tyne, UK
2 Population Health Sciences Institute, Newcastle upon Tyne, UK

276 Two techniques,one goal: a prospective study on early postoperative outcomes between stapled and hand-sewn gastrojejunostomy in distal gastric cancer

Joty Syeda Mehbuba 1
Alam Ferdous 1
Sunny SM Syyed-Ul-Alam 1
Saiyara Noshin 2
Sarker Ashok Kumar 3
1 Bangladesh Medical University, Dhaka, Bangladesh
2 Derby Hospitals NHS Foundation Trust, Derby, UK
3 Enam Medical College Hospital, Dhaka, Bangladesh

297 Bucks bites: a retrospective review of a single centre experience of bites to the hand

Lingamanaicker Vidhya
Bricogne Christopher
Lim Colleen
Shirley Rebecca
Stoke Mandeville Hospital, Buckinghamshire, UK

316 Mean serum PSA levels before and after TURP: insights from BPH patients.

Naz Kanwal 1 2
Seetlani Vikram 2
Hussain Mushtaq 3 2
1 Russells Hall Hospital, Dudley, UK
2 Sindh Institute of Urology and Transplantation, Karachi, Pakistan
3 Queen Elizabeth Hospital Birmingham, Birmingham, UK

332 Exploring patient narratives on diaphragm dysfunction: a social media listening study

Clayton Sofia G Villavicencio 1 2
Bajaj Amrita 3
Evans Rachael A 4 5
Caruana Edward J 2 5
1 University of La Laguna Medical School, La Laguna, Spain
2 Thoracic Surgery, Glenfield Hospital, Leicester, UK
3 Radiology, Glenfield Hospital, Leicester, UK
4 Department of Respiratory Sciences, University of Leicester, Leicester, UK
5 Institute for Lung Health, NIHR Leicester BRC, Leicester, UK

338 Technological versus clinical monitoring in breast reconstruction: a systematic review and meta-analysis

Dhannoon Amenah 1
Wong Zhen Yu 1
Zargaran Alexander 2
Mosahebi Afshin 2
1 Welsh Centre for Burns & Plastic Surgery, Morriston Hospital, Swansea, UK, Swansea, UK
2 Royal Free London NHS Foundation Trust, London, UK

358 Complete versus incomplete percutaneous oblique distal closing wedge osteotomy for bunionette (Tailor’s Bunion) deformity correction: a comparative study

Mehrotra Sanjana 1
Newton Ayla 2
Ahamed Mohamed Wasim Shaffe 3
Wickramarachchi Lilanthi 3
Yousef Mohamed Sayed 3
Lam Peter 4
Lewis Thomas L 3
Ray Robbie 3
1 University of Sheffield, Sheffield, UK
2 King’s Foot and Ankle Unit, King’s College Hospital NHS Foundation Trust, UK, London, UK
3 King’s Foot and Ankle Unit, King’s College NHS Foundation Trust, UK, London, UK
4 Orthopaedics and Arthritis Specialist Centre, Sydney, Australia, Sydney, Australia

407 Reamed vs unreamed intramedullary nailing for fragility fractures of the proximal femur – a retrospective study

Mehrotra Sanjana 1
Pandya Chiraag 2
Dackombe Meera 2
Iftinca Dorin 2
Lincoln Henry 2
Virgo Kathryn 2
Wembridge Kevin 2
1 Sheffield Medical School, University of Sheffield, Sheffield, UK
2 Rotherham District General Hospital, Rotherham, UK

425 Ten-year outcomes of bladder stone management at a UK tertiary centre: a retrospective data analysis

Shetty Neehar 1
Xu Joshua 2
Croghan Stephanie 1
Page Tobias 1
Rogers Alistair 1
1 The Freeman Hospital, Newcastle, UK
2 Newcastle University, Newcastle, UK

433 Safe and feasible in a low-income setting: complications of ultrasound-guided PCN assessed with the Modified Clavien System

Hussain Mushtaq 1 2
Iqbal Muhammad Danial 2
Waleed Ahmad 2
Gazder Tanzeel-Ur-Rahman 2
1 Queen Elizabeth Hospital, Birmingham, UK
2 Sindh Institute of Urology and Transplantation, Karachi, Pakistan

446 Evaluating the role of adjuvant chemotherapy in post-resection outcomes of pancreatic cancer

Garcia Marta Chacon 1
Theivendrampillai Thrshegan 1
Frampton Adam 1 2
1 Department of HPB Surgery, Royal Surrey NHS Foundation Trust, Guildford, UK
2 University of Surrey, Guildford, UK

456 Does prior endoscopic management influence success of anterior urethroplasty?

Hussain Mushtaq 1 2
Naz Kanwal 2
Abidi Syed Saeed 2
1 Queen Elizabeth Hospital, Birmingham, Pakistan
2 Sindh Institute of Urology and Transplantation, Karachi, Pakistan

Subjects: Collaborative Research

300 Acute otitis media in children with cleft lip and/or palate: a narrative synthesis of the literature

Mitra Kurchi 1 2
Nair Sanjana 3
Kazi Maliha 1
Butterworth Sophie 4
Powell Jason 2 1
1 Department of Paediatric Otolaryngology, Great North Children’s Hospital, The Newcastle upon Tyne Hospitals NHS Foundation Trust, Newcastle-upon Tyne, UK
2 Translational and Clinical Research Institute, Faculty of Medical Sciences, Newcastle University, Newcastle-upon Tyne, UK
3 Department of Acute Medicine, Sunderland Royal Hospital, Sunderland, UK
4 Department of Plastic Surgery, Great North Children’s Hospital, The Newcastle upon Tyne Hospitals NHS Foundation Trust, Newcastle-upon Tyne, UK

Subjects: Early-Stage Innovation

11 Pre-MDT flow project

Akhtar Amina
Andreyev Daniel
Govindaraju Krishmitha Jothy
McCulloch Pauline
Thin Noel
Dvorkin Lee
Navaratnam Romesh
North Middlesex Hospital, London, UK

27 Forging a new frontier in neurosurgical research: a replicable leadership model for building research capacity in a low- and middle-income country setting

Saghebdoust Sajjad 1 2
Jouzdani Ali Fathi 2
Zahmatkesh Mohammad Reza Rouhbakhsh 2
Zare Reza 2
1 Department of Neurosurgery, Queen's Hospital, Barking Havering and Redbridge University Hospitals NHS Trust, Romford, UK
2 Department of Neurosurgery, Razavi Hospital, Mashhad, Iran, Islamic Republic of

139 Early-stage innovation: artificial intelligence in flap perfusion assessment and monitoring

Shakir Mohammed
Manchester NHS Foundation Trust, Manchester, UK

176 Evaluating the role of simulation-based medical teaching on teaching core competencies in undergraduate surgical education: interim results of a randomised control trial

Brennan Aimee 1 2
Kennedy Czara 1 2
Coyle Hilary 1
McEntee Philip 2
Egan Katherine 1 2
Cronin John 3
Butt Waqas 1 2
Heneghan Helen 1 2
1 School of Medicine, University College Dublin, Dublin, Ireland
2 Department of Surgery, St Vincent's University Hospital, Dublin, Ireland
3 St Vincent's University Hospital, Dublin, Ireland

189 Pilot evaluation of an AI-powered medication assistant for detecting drug–drug interactions in hospitalised patients

Singh Roshan Ajeet
Sivakumar Wymin
Hennessy Derek B
Mercy University Hospital, Cork, Ireland

252 From simulation to surgery: validating the chicken sternal cartilage model as a transitional tool in rhinoplasty training

Kiew Cheuk Ying Kyleen 1
Shah Anuska 1
Singh Rananjay 1
Atherton Duncan 2
1 Imperial College London, London, UK
2 Evelina London Children's Hospital, London, UK

261 Progressive, low-cost simulation models to enhance undergraduate microsurgery exposure: a student-led conference

Singh Rananjay 1
Kiew Cheuk Ying Kyleen 1
Mahmood Ameen 1
Shah Anuska 1
Zaidi Hasan 1
Kutaiman Tarek 1
Gokani Vimal 2
1 School of Medicine, Imperial College London, London, UK
2 Department of Plastic and Reconstructive Surgery, Imperial College Healthcare NHS Trust, London, UK

283 Improving the induction experience for plastic surgery resident doctors through digital resources

Nowrin Maysha
McMillan Angus
Clay William
Jivan Sharmila
Mid Yorkshire NHS Teaching Trust, Wakefield, UK

296 Scalpel meet DIY: bridging the gap between simulation and surgical fidelity

Fernandez Nicole Salgado 1
Piozzi Guglielmo 2
Khan Jim 2
1 University of Buckingham, Buckingham, UK
2 Portsmouth Hospital University NHS Trust, Portsmouth, UK

321 Novel method for successful placement of double lumen urodynamics catheters in men who are difficult to catheterise

Olaitan Oluwabukola
Ravindra Pravisha
Rai Jaskarn
Leicester General Hospital, Leicester, UK

364 Neuroendoscopic trans-ventricular cyst drainage prior to tumour resection using a two-staged approach for cystic craniopharyngioma: a case-based review

Javid Saeed
Queen's Hospital, Romford, UK

384 Adaptation of glue injection needle for haemorrhoidal sclerotherapy

Batra Paras
Sharma Abhiram
Manchester University Foundation Trust, Manchester, UK

387 Has Optilume drug-coated balloon urethral dilatation for recurrent urethral strictures expanded our treatment options: a narrow follow-up snapshot

Shetty Neehar
Rizvi Ishtiakul
Barclay Jonathan
The Freeman Hospital, Newcastle, UK

477 Hearing preservation surgeries in management of endolymphatic sac decompression – a dying surgery in ENT?

Menezes Sarah
Basavaraj Sreeshyla
St Mary's Hospital, Isle of Wight, UK

Subjects: Global Surgery

20 Transformational leadership and team engagement in the neurosurgical operating theatre: a prospective, video-recorded observational study

Saghebdoust Sajjad 1 2
Jouzdani Ali Fathi 2
Soltani Ghasem 3
Zare Reza 2
1 Department of Neurosurgery, Queen's Hospital, Barking Havering and Redbridge University Hospitals NHS Trust, Romford, UK
2 Department of Neurosurgery, Razavi Hospital, Mashhad, Iran, Islamic Republic of
3 Department of Anesthesiology, Razavi Hospital, Mashhad, Iran, Islamic Republic of

21 Unmasking post-operative delirium in elderly neurosurgical patients: a closed-loop clinical audit to improve screening at a tertiary teaching hospital

Saghebdoust Sajjad 1 2
Jouzdani Ali Fathi 2
Zahmatkesh Mohammad Reza Rouhbakhsh 2
Zare Reza 2
1 Department of Neurosurgery, Queen's Hospital, Barking Havering and Redbridge University Hospitals NHS Trust, Romford, UK
2 Department of Neurosurgery, Taleghani Hospital, Mashhad University of Medical Sciences, Mashhad, Iran, Islamic Republic of

33 Restrictive bariatric surgery in focus: what’s next for endoscopic sleeve gastroplasty and laparoscopic sleeve gastrectomy

Tripathi Swapnil 1 2
Ray Avinash 3
Sinha Yash 1
Noormohamed Saleem 1
Reid Alastair 1
1 Worcestershire Acute Hospital NHS Trust, Worcester, UK
2 Armed Forces Medical College, Pune, India
3 Army Medical Corps/ Indian Air Force, Gwalior, India

51 Bariatric surgeries outcomes on geriatric age group: a retrospective study in Egypt

Fahem Michael 1
Zaki Anasimone 2
Antonious Cathrine 3
abdelghani Mohamed 2
Ayoub Peter 4
1 Al Kasr Al Aini University Hospitals, Cairo, Egypt
2 Al Mokattam Health Insurance Hospital, Cairo, Egypt
3 Helwan University Hospitals, Cairo, Egypt
4 Tanta University Hospitals, Tanta, Egypt

174 The factors influencing the conversion from laparoscopic to open cholecystectomy at Muhimbili National Hospital

Mohamed Esra
University of Medical Science and Technology, Al Khartoum, Sudan. Aswan Teaching Hospital, Aswan, Egypt

242 Pelvic floor reconstruction with retroverted uterus after extralevator abdominoperineal excision for rectal carcinoma: cost effective technique in select women useful in low- and middle-income countries

Visweswaraiah Shiva Pratap Unnenahalli 1 2
Varghese Gigi 1 2
1 Christian Medical College, Vellore, India
2 University Hospital of North Midlands, Stoke on Trent, UK

324 Diagnostic pitfalls in endemic regions: MEST mimicking hydatid disease

Hussain Mushtaq 1 2
Naz Kanwal 2
Mahar Naveed 2
1 Queen Elizabeth Hospital, Birmingham, UK
2 Sindh Institute of Urology and Transplantation, Karachi, Pakistan

436 Gender representation among surgeons working in low-income countries affected by conflict and war: a scoping review

Wardak Abdul Baseer MBBS MD 1
Basu Eilene 2
1 Gender equity initiative in global surgery (GEIGS), Peshawar, Pakistan
2 Gender equity initiative in global surgery (GEIGS), Boston, USA

Subjects: QI and Audit

2 Assessing the yield: investigating the incidence of significant findings on flexible sigmoidoscopies in patients with PR bleeding

Quimpo Jessica 1
Syed Muhammadtaaha 1
Iftikhar Umar 1
Rushbrook Sophie 2
Rane Aditi 3
Kamel Faddy 1
1 Croydon University Hospital, London, UK
2 City St George's University, London, UK
3 University College Hospitals London, London, UK

10 The abscess pathway and how much it cost the NHS in a single centre

Ng Yuki Julius 1 2
Muhammad Alshibshoubi 1
Rossi Chiara 1
Tariq Maha 1
Carney Eleanor 1
Butcher Katrina 1
1 University Hospital Bristol and Weston, Weston-Super-Mare, UK
2 International Association of Student Surgical Society, Cape Town, South Africa

24 Enhancing patient and family education in post-operative neurosurgical care: a structured quality improvement initiative to reduce complications and improve satisfaction at a tertiary care center

Saghebdoust Sajjad 1 2
Jouzdani Ali Fathi 2
Zahmatkesh Mohammad Reza Rouhbakhsh 2
Zare Reza 2
1 Department of Neurosurgery, Queen's Hospital, Barking Havering and Redbridge University Hospitals NHS Trust, Romford, UK
2 Department of Neurosurgery, Razavi Hospital, Mashhad, Iran, Islamic Republic of

25 An improving quality in healthcare initiative: enhancing interdisciplinary communication during neurosurgical patient handovers using a standardised protocol

Saghebdoust Sajjad 1 2
Jouzdani Ali Fathi 2
Saghebdoust Soheil 3
Zare Reza 2
1 Department of Neurosurgery, Queen's Hospital, Barking Havering and Redbridge University Hospitals NHS Trust, Romford, UK
2 Department of Neurosurgery, Taleghani Teaching Hospital, Mashhad University of Medical Sciences, Mashhad, Iran, Islamic Republic of
3 Department of Physics and Astronomy, University of Manitoba, Winnipeg, Canada

28 Is it still PHPT? Evaluating normohormonal presentations in severe hypercalcaemia

Raven-Gregg Timia
Sabah Yousuf
Micu Vlad
Egan Richard
Swansea Bay University Healthboard, Swansea, UK

30 Optimising outcomes in geriatric emergency laparotomy: a quality improvement initiative for consistent COTE consultant review

Maula Kulsum 1
Vu John 1
Thompson-Reil Colette 1
Miller Roy 2
1 East Suffolk and North Essex NHS Foundation Trust, Colchester, UK
2 East Suffolk and North Essex NHS Foundation Trust, Colchester, UK

34 Saving time for ENT doctors with innovative emergency on-call bag

Eves Peter
Hassan Syed Wajih Ul
Southern Health and Social Care Trust, Criagavon, UK

37 Circumcised foreskin – to routinely send or not send for histopathological analysis?

Ojo Charles
McAuley Laura
Southern Health and Social Care Trust, Portadown & Newry, UK

70 Assessing referral appropriateness for torus (buckle) and minimally displaced clavicle fractures to a paediatric virtual fracture clinic

Roberts Edward 1
Deriu Laura 2
1 University of Leeds, School of Medicine, Leeds, UK
2 Leeds Teaching Hospitals Trust, Leeds, UK

103 Comparison of ward round notes before and after the introduction of an electronic note system

Fiorentini Gianluca
Wharry Rebecca
Stokes Michael
Altnagelvin Area Hospital, Derry, UK

148 Audit of testicular torsion management, comparison with BAUS FIX-IT guidance

Shaheen Milad
Murray Toby
Oluwole-ojo Akinlolu
Papikinos Panagiotis
East Surrey Hospital, Redhill, UK

151 The financial and strategic impact of accurate coding in elective orthopaedic surgery: a retrospective audit

Usman Rukayya 1
Hadi Hosain 1
Metin Canan 1
Nwankwo Ezidinma 1
Piciorea Mihaela 1
Samir Mina 1
Mahmood Abid 2
Janipireddy Satish 1
Kurer Mike 1
1 North Middlesex University Hospital, London, UK
2 Maidstone & Tunbridge Wells NHS Trust, Kent, UK

152 Closed-loop audit to improve trauma care by adhering to the guidelines for attendance and documentation of trauma calls in one of London’s major trauma centres

Linn Htoo Htet
Pages Clara Calero
Manoharasundaram Kriisan
Frith Daniel
Imperial College Healthcare NHS Trust, London, UK

154 A closed-loop clinical audit to improve perioperative dexamethasone stewardship in supratentorial tumour surgery

Saghebdoust Sajjad 1 2
Saghebdoust Soheil 3
Jouzdani Ali Fathi 1
Zare Reza 1
1 Department of Neurosurgery, Razavi Hospital, Mashhad, Iran, Islamic Republic of
2 Department of Neurosurgery, Queen's Hospital, Barking Havering and Redbridge University Hospitals NHS Trust, Romford, UK
3 Department of Physics and Astronomy, University of Manitoba, Winnipeg, Canada

158 A quality improvement project to improve ward round documentation using the Heidi health application

Qamar Amna
Kelly Maired
Maweni Robert
Abdul-Hamid Ayeshah
Oxford University Hospitals NHS Trust, Oxford, UK

159 Improving operative note documentation through low-cost interventions: a closed-loop audit in general surgery

Agarwal Anurag
Balasubaramaniam Vignesh
Hassan Umair
Abdullah Nik
Betsi Cadwaladr University Health Board, Bangor, UK

160 Evaluating BOAST 4 compliance during the establishment of a regional plastic surgery unit: a two-cycle audit

Kiwan Omar 1
Rafie Arash 2
Al-Kalbani Mohammed 1
Nasim Mazhar 3
Yao Susie 3
1 University of Manchester, Manchester, UK
2 Manchester University NHS Foundation Trust, Manchester, UK
3 Northern Care Alliance NHS Foundation Trust, Salford, UK

162 Improving safeguarding in paediatric burns: implementing the BuRN tool in outpatient clinics

Thomas Caitlyn
Hartrick Olivia
Bowman Charlotte
Willett Hannah
Avent Zoe
Connett Sophie
Murray Alexandra
Stoke Mandeville Hospital, Aylesbury, UK

167 Assessing general surgery admissions - opportunities for next day acute surgical unit review

Mahmoood Dawood
Mahmood Akhtar
Cromwell Paul
Galway University Hospital, Galway, Ireland

178 Sustainable surgery in practice: an audit of reusable vs single-use gowns

Al-kalbani Mohammed 1
Munro Tyler 2
Khouja Adam El 3
Shekrobat Ayah Assadi 3
Kiwan Omar 1
1 University of Manchester, Manchester, UK
2 Bradford Teaching Hospitals NHS Foundation Trust, Manchester, UK
3 Wrightington, Wigan and Leigh Teaching Hospitals NHS Foundation Trust, Wigan, UK

179 Audit of flexor tendon injury management: a three-cycle review of compliance with BSSH guidelines

Eaton Daniel
Sebastiao Alexandra
Cook Hannah
Giwa Lola
Kyprianou Katerina
Sharma Vikram
Akhavani Mo
Royal Free NHS Foundation Trust, London, UK

182 Landmark-guided knee injections: a cost-effective alternative to ultrasound in outpatient clinics

Baskar Sangeetha
Pattnaik Saphalya
Lewisham and Greenwich NHS Trust, London, UK

185 Radiological and functional outcomes after fixation: the relevance of Soong grades

Pattnaik Saphalya 1
Khalid Mohamed 1
Sidhu Gur Aziz Singh 2
1 Lewisham and Greenwich NHS Trust, London, UK
2 King College Hospital NHS Trust, London, UK

187 Periprosthetic joint infections in arthroplasty: local audit against national standards

Pattnaik Saphalya 1
Gawad Mothana 1
Sidhu Gur Aziz Singh 2
1 Lewisham and Greenwich NHS Trust, London, UK
2 Kings College Hospital NHS Trust, London, UK

192 An Audit evaluating environmental and financial impacts of intra-operative intravenous (IV) paracetamol use, and implementation of a policy document to transition from intra-operative IV paracetamol use to pre-operative oral paracetamol use, for elective surgical patients at St Vincent’s Hospital, Sydney

Zulueta Sofia
St Vincent’s Hospital Sydney, Sydney, Australia

193 A scalable consultant-led pathway for paediatric burns: one-week review with routine laser Doppler flowmetry

Floros Georgios Rafail
Pinderfields Hospital NHS Trust, Wakefield, UK

195 Optimising indications for foreskin histopathology post-circumcision: a quality improvement initiative

Bochinski Antoni
Ray Rishabh
Keeling Ellie
Rai Jaskarn
University Hospitals of Leicester NHS Trust, Leicester, UK

196 STEP-up for surgery: a simulation-based teaching and enrichment programme for resident doctors starting surgical rotations

Slater Grace
Watson Katherine
Iftimie-Nastase Ioana
Mid Yorkshire Teaching NHS Trust, Wakefield, UK

202 Do we adequately counsel patients on the substantial consequences of radical pelvic cancer surgery for bladder, bowel and sexual function?

Ditri Daria 1
Pungpapong Gunn 1
Ghaem-Maghami Sadaf 1 2
Drake Marcus 1 2
1 Imperial College London, London, UK
2 Imperial College Healthcare Trust, London, UK

205 Audit of ePROMs uptake and patient-reported outcomes following penectomy and nodal surgery

Chowdhury Mahin
Hodgson Clare
Sangar Vijay
The Christie Hospital, Manchester, UK

212 Evaluation of the knowledge and confidence of foundation doctors in catheter and drain management

Zhang Qian
Rabinowitz Joshua
Royal Free Hospital, London, UK

217 Evaluating colonoscopy rescope requests: service burden and clinical justification

Joseph Debora
Stubbs Benjamin
Dorset County Hospital, Dorchester, UK

224 FAST in name, slow in practice: a QIP to improve trauma care in line with ATLS/ETC

Gurung Ganga
Singh Christopher
Landolfi Catello
Hereford County Hospital, Hereford, UK

226 Reducing inappropriate post-operative laxative prescribing following elective colorectal surgery: a clinical audit

Jha Nikita
Ugur Suha
Leeper Victoria
Talwar Anjay
Dorset County Hospital, Dorchester, UK

231 Evaluation of clinical practise in relation to BOAST 11 guidelines: supracondylar humeral fractures in children

Khan Arif 1 2
Bhatti Zarafshan 3
1 Wirral University Teaching Hospital, Wirral, UK
2 Northern Care Alliance NHS Foundation Trust, Manchester, UK
3 Northern Care Alliance NHS Foudation Trust, Manchester, UK

239 Evaluating the presence of recycling information on medical packaging

Tittawella Ashwini
Bohonos Colton
McDermott Jack
Hennessey Derek
Mercy University Hospital, Cork, Ireland

248 Beyond the orchidectomy: benchmarking testis cancer care against ANZUP standards in a high-volume Australian centre

Grudgings Penny
Al-Khanaty Abdullah
Lasry Beatrice Hornstein
Peter MacCallum Cancer Center, Melbourne, Australia

251 Optimising the transurethral resection of bladder tumour (TURBT) pathway: introducing a clinician-led scheduling system

McGeoghan Lauren
Morrow Jessica
Curry David
Keane Patrick
Belfast City Hospital, Belfast, UK

253 Shaping surgical futures: insights into specialty life through the Surgical Specialty Spotlight Series, a global teaching initiative for tomorrow’s surgeons

Subbiah Praveen 1
Vinoo Akshay 2
Uddin Aalia 3
1 Hereford County Hospital (Wye Valley NHS Trust), Hereford, UK
2 Liverpool University Hospital NHS Foundation Trust, Liverpool, UK
3 Queen Victoria Hospital NHS Foundation Trust, East Grinstead, UK

254 Day-case solutions: re-designing paediatric trauma services

Zahid Abra
Khincha Priyatma
McSweeney Jared
Bedford James
Rivers Clare
MFT, Manchester, UK

259 Improving practice in acute diverticulitis: an audit of antibiotic use and endoscopic follow up against ESCP guidelines

Balamurugan G 1
Innes Ailsa 2
Heads Mark 1
Peters Francesca 2
Singh Daya 1
1 South Tyneside and Sunderland NHS Foundation Trust, South Shields, UK
2 Northumbria Healthcare NHS Foundation Trust, Newcastle upon Tyne, UK

264 Boosting theatre performance and productivity in elective surgery through audit and feedback

Balamurugan G 1
Sayyadi Layla 1
Taswar Rimshah 1
Tomlin Jamie 2
Singh Daya 1
1 South Tyneside and Sunderland NHS Foundation Trust, South Shields, UK
2 The Newcastle Upon Tyne Hospitals NHS Foundation Trust, Newcastle upon Tyne, UK

270 Necessity Of surgical revascularization of the diagonal artery during isolated CABG: outcomes in patients managed without diagonal grafting

Gannon Eiran
Miskell Machaela
Soo Alan
University Hospital Galway, Galway, Ireland

291 A prospective closed-loop audit of patient-reported experience measures (PREMs) in urodynamic studies

Subbiah Praveen 1
Qasrina Farah Nurin 2
Pai Aakash 2
1 Hereford County Hospital, Hereford, UK
2 Northampton General Hospital NHS Trust, Northampton, UK

293 Improving catheter documentation in a tertiary urological centre: a quality improvement project

MacKay Murray
NHS Lothian, Edinburgh, UK

298 Assessing staff attitudes towards sustainability in operating theatres

Bhargava Charvi 1
Bhargava Seema 2
Marghade Pallavi 2
1 Anglia Ruskin University, Chelmsford, UK
2 Basildon University Hospital, Basildon, UK

301 Implementing a change of gloves and instruments prior to abdominal wound closure in general surgery to reduce rates of surgical site infection

Purser Lorcan
Chesterfield Royal NHS Foundation Trust, Chesterfield, UK

303 Audit of the oesophago-gastric cancer diagnostic pathway at UHDB NHS foundation trust

Sohrabi Zohal
Royal Derby Hospital, Derby, UK

305 Virtual fracture clinic improves BOAST compliance, patient outcomes, and delivers cost savings in lower socio-economic areas

Khan Rania 1
Hadi Hosain 1
Mahmood Abid 2
Backer Afnan 1
Khan Shahnawaz 1
Atkinson Henry D 1
1 North Middlesex Hospital, London, UK
2 Maidstone and Tunbridge Wells NHS Trust, Kent, UK

309 Open abdomen management in trauma and emergency surgery: a two-year audit

Deshraju Aslesha
Freeman Orla
Linn Htoo
Navaratne Lalin
Imperial College Healthcare NHS Trust, London, UK

312 Reducing surgical site infections: SSI bundle implementation at Wrexham Maelor Hospital

Battersby C.
Pereira M.
Ravindran T. A
- S.
Sogaulu O.
Idama D.
Garbutt Y.
Department of General Surgery, Wrexham Maelor Hospital, Wrexham, UK

315 More is better

Menon Rahul
Smith Louise
Ansell Holly
Menon Achyuth
King's Mill Hospital, Sutton-In-Ashfield, UK

322 Audit of microbiological analysis and clinical utilisation of organ perfusion fluid in renal transplantation

Amo-Afful Samuel 1
Adelmageed Ahmed 2
Uwechue Raphael 2
1 Isle of Wight NHS Trust, Isle of Wight, UK
2 Queen Alexandra Hospital, Portsmouth, UK

327 Apixaban use peri-operatively in orthopaedics

Jemmett Hermione
Saleh Nivar
The Royal infirmary of Edinburgh, Edinburgh, UK

329 Beyond the scan: accuracy and limitations of mpMRI in prostate cancer diagnosis

Naz Kanwal 1
Adenipekun Ayokunle 1
Hussain Mushtaq 2
Ubee Sarvpreet 1
Birring Amerdip 1
Shafik Adel 1
1 Russells Hall Hospital, Dudley, UK
2 QueenElizabeth Hospital, Birmingham, UK

331 Enhancing handover and continuity of care: a closed-loop audit of digital documentation in thoracic surgery at a teaching hospital

Aziz Amirah Adlina Binti Abdul
Qsous Ghaith
Akhter Alina
Azam Hariz Azhari Bin Nor
Nottingham University Hospital NHS Trust, Nottingham, UK

344 LIVER study – liver injury vigilance, evaluation and response: evaluating liver trauma surveillance in high-grade injuries: a retrospective analysis of compliance, intervention rates and outcomes at St Mary’s major trauma centre

Karagiannidis Georgios 1
Banham Evie 2
1 Imperial College London, London, UK
2 ESNEFT, Ipswich, UK

346 From white-top to red-top: a simple change improving urine culture accuracy in ureteroscopy

Collangettes Maelle
Dhariwal Randeep
Arumuham Vimoshan
University College London Hospitals NHS Foundation Trust, London, UK

347 Standardising flexor tendon repair documentation and enhancing patient communication: a two-cycle audit and qi project

Phillips Harry
Addenbrookes Hospital, Cambridge, UK

350 Low diagnostic yield of the two-week wait pathway for testicular cancer: is there a role for pre-referral ultrasound?

Hussain Mushtaq 1
Naz Kanwal 2
Sarkar Debashis 1
1 Queen Elizabeth Hospital, Birmingham, UK
2 Russells Hall Hospital, Dudley, UK

362 Optimizing hand trauma management in a tertiary NHS plastic surgery department: a quality improvement initiative aligned with UK prevalence data and BSSH guidelines

Dey Shimul
University Hospital Coventry and Warwickshire, Coventry, UK

373 Treatment outcomes following intravesical onabotulinum toxin a (Botox) Injection: a retrospective cohort study

Abolanle Philip
Richards Thomas
Hussain Paulette
Seymour Coral
Saunders Rebecca
Eylert Maike
Aneurin Bevan University Health Board, Newport, UK

383 Risk stratification post radical nephrectomy: an evaluation of Leibovich Score in clear cell renal cell carcinoma (ccRCC) report

Tang Kevin Xuan Hong
Alnoomani Mohamed
Ibrahim Mohamed
Allat Omar
Emara Amr
Hampshire Hospitals Foundation Trust, Winchester, UK

393 a lower limb free flap enhanced dangling protocol initiative at a major trauma centre: introducing LEAP-ER (Lower Limb Enhanced Accelerated Pathway-Early Recovery) protocol

Dey Shimul
Hossain Rehnuma
Wallace David
University Hospital Coventry and Warwickshire, Coventry, UK

395 Reducing delays to PEG insertion in postoperative head and neck cancer patients: a quality improvement project at Oxford University Hospitals

Malik Danish
Sivanathan Shayndhan
Shaw Sarah
Prabhu Satheesh
Oxford University Hospitals, Oxford, UK

412 Reducing catheter duration to improve recovery in hip fracture patients: a two-cycle audit

White Malin
Dimitry Jonathan
Diab Ahmad
Chatterjee Suharto
Pilgrim Hospital, Boston, UK

416 Bloodless victory: a two-cycle audit to reduce the use of routine group and save testing in day case laparoscopic cholecystectomy

Sundarraj Jeeva Karuniya
Watt Eve
Mapara Rahee
McIlveen Erin
NHS GJNH, Glasgow, UK

418 Leveraging electronic patient record technology to optimise Oakland score use in lower gastrointestinal bleeding

Khan Faraaz
Emmanuel Andrew
King's College Hospital, London, UK

419 Optimising on-call workflow in general surgery through EPIC-integrated handover Tools

Khan Faraaz
Al-Akash Musallam
King's College Hospital, London, UK

421 An audit of the management of tetanus-prone wounds in hand trauma patients at a UK plastic surgery unit

Lingamanaicker Vidhya
Stoke Mandeville Hospital, Buckinghamshire, UK. St George's Hospital, London, UK

430 Complications and diagnostic accuracy of local anaesthetic transperineal prostate biopsy: a 6-month single-centre audit

Derex-Briggs Jasmine
Worthing Hospital, Worthing, UK. Allaudin Issa, Worthing, UK. Freya Miller, Worthing, UK. William Britnell, Worthing, UK

432 Patient experience of urodynamic studies: a first-cycle audit against BAUS standards

Sriranganathan Sarankan
Kar Irfan
Hasan Adel Shaikh
Alomari Mohammad
East and North Hertfordshire NHS Trust, Stevenage, UK

Subjects: Robotics and Digital Surgery

78 Early outcomes of robotic radical cystectomy: initial single-centre experience

McAdam Jonathan
McAdam Andrew
Belfast City Hospital, Belfast, UK

86 Optimizing robotic pyeloplasty: a QIP of an efficient elective service model in a district general hospital

Bobby Ashwin Paul
Krishnan Claire Luke
Hosny Khaled
East Lancashire Hospitals NHS Foundation Trust, Blackburn, UK

314 The Scottish robotic colorectal surgery pilot training programme (RCTP) – reflections on implementing a collaborative programme specific for colorectal trainees

Abduldaiem Yousef
Akbar Muhammed Bilal
Vera Vithya
Shah Adarsh
Carden Claire
NHS Tayside, Dundee, UK

Subjects: Systematic Review and Meta-Analyses

47 Mesh repair versus no-mesh repair for the management of acute and elective femoral hernia: a systematic review and meta-analysis comparing the perioperative outcomes

Abdalgany Ahmed
Malkawi Hashem
Abdelgawad Mohammed Abdelgawa
Najjar Hussein
University hospital Sussex NHS Foundation Trust, Brighton, UK

91 Management of carpal tunnel syndrome: a network meta-analysis

Challoumas Dimitris
Edgar Nathan
Barrett Brendan
Ihlbrock Emily
Ibekewe Nachika
Ahmad Ali
Tham Alexander
Munn David
Polzer Stella
Sinnerton Rob
University of Glasgow, Glasgow, UK

97 Exploring the new horizon: a systematic review of artificial intelligence (AI) in urology

Bhowmik Prasenjit
Osman Banan
Urology Department, Heartlands Hospital, University Hospitals Birmingham NHS Foundation Trust, Birmingham, UK

137 Topical stem cell sprays and suspensions for post-burn wound healing: a systematic review of emerging regenerative therapies

Shakir Mohammed
University College London, London, UK

141 Evaluating the effectiveness of personalised video-based coaching for the acquisition of surgical skills in minimally invasive surgery; a systematic review and meta-analysis

Bery Peter
University of East Anglia, Norwich, UK

145 Exploring the role of perioperative dexamethasone in pancreaticoduodenectomy: helpful adjunct or hidden risk? A meta-analysis

Shehzad Mishkat 1
Sultan Syed Muhammad Moaaz Bin 1
Imran Muhammad Anzal 1
Nizami Aaes 2
1 Dow University of Health Sciences, Karachi, Pakistan
2 Karachi Medical and Dental College, Karachi, Pakistan

175 Clinical outcomes of hip hemiarthroplasty in patients with Parkinson’s Disease following femoral neck fractures: a systematic review

Roche Eimear
Murphy Colin
Galway University Hospital, Galway, Ireland

181 Absorbable versus non-absorbable sutures for facial skin cancer closure: a systematic review of patient-reported outcome measures

Kaur Noor 1
Mandal Anirban 2
1 University of Liverpool, Liverpool, UK
2 St Helens and Knowsley Trust, Prescot, UK

268 The role of pre-operative systemic corticosteroids in functional endoscopic sinus surgery: a systematic review and meta-analysis

Wanniarachchi Kujani 1
Morris James 1
Patel Dhruv 1
Omer Tareq 1
Yap Darren 2
1 University of Cambridge, Cambridge, UK
2 Oxford University Hospitals, Oxford, UK

278 The role of testosterone in lower urinary tract symptoms: help or harm? – systematic review

Bhowmik Prasenjit
Osman Banan
Heartlands Hospital, University Hospitals of Birmingham NHS Foundation Trust, Birmingham, UK

317 The impact of artificial intelligence on clinical outcomes in general surgery: a systematic review and meta-analysis

Siddique Muhammad Harris
MMUH, Smethwick, UK

330 Systematic review of strategies to minimise extraction site pain after laparoscopic sleeve gastrectomy

Nahid Mahmudul Hasan 1
Noor Mehnaj 2
Padmanaban Aravind Adarsh 3
Hossain Naveed 4
Hossain Tanvir 3
1 University Hospital Leicester, Leicester, UK
2 University Hospital of Southampton, Wessex, UK
3 University Hospitals Birmingham, Birmingham, UK
4 Imperial College London, London, UK

334 Impact of reduction mammaplasty on body mass index: a systematic review and meta-analysis

Fong Wei Yu 1
Wong Zhen Yu 2
Faderani Ryan 3
Kanapathy Muholan 3
Mosahebi Afshin 3
1 Whiston Hospital, Prescot Merseyside, UK
2 Morriston Hospital, Swansea, UK
3 UCL, London, UK

341 Operative versus conservative management of nailbed injuries: a systematic review and meta-analysis

Dhannoon Amenah 1
Wong Zhen Yu 1
Zargaran Alexander 2
Mosahebi Afshin 2
1 Welsh Centre for Burns & Plastic Surgery, Morriston Hospital, Swansea, UK, Swansea, UK
2 Royal Free London NHS Foundation Trust, London, UK

357 CONSORT compliance in prostatectomy randomised control trials: a systematic review

Subbiah Praveen 1
Khan Gul Rukh 2
Mitchell Sebastian 3
Jameel Mohammed 4
1 Hereford County Hospital, hereford, UK
2 West Hertfordshire Teaching Hospitals NHS Trust, Watford, UK
3 St Johns Hospital, Howden, UK
4 East Lancashire Hospital NHS Trust, Blackburn, UK

388 Dual mobility versus large femoral head in primary and revision total hip arthroplasty: a meta-analysis of outcomes

Ibrahim Abdelrahman
Selim Amr
Dwebeng Christina
Choudhary Zain
Singal Sachin
Ahmed Mohamed
Khamdan Khadija
Al-musabi Musab
Thomas Geraint
University Hospitals of North Midlands NHS trust, Stoke on Trent, UK

390 The impact of smoking on outcomes following anterior cruciate ligament reconstruction: a systematic review and meta-analysis

Ibrahim Abdelrahman
al-musabi Musab
Kabariti Rakan
Tahir Muaaz
Wade Roger
University Hospitals of North Midlands NHS Trust, Stoke on Trent, UK

420 Laparoscopic versus endoscopic strategies for common bile duct stones: a systematic review and network meta-analysis of randomised trials

Shah Ali Akbar
London North West University NHS Trust, London, UK

